# Distinct roles for LTalpha3 and LTalpha1beta2 produced by B cells contribute to their multi-faceted impact on ileitis

**DOI:** 10.21203/rs.3.rs-3962916/v1

**Published:** 2024-02-26

**Authors:** Gwendalyn Randolph, Emma Erlich, Rafael Czepielewski, Rachael Field, Taylor Dunning, Leila Saleh, Mark Hoofnagle, Alexei Tumanov, Farshid Guilak, Jonathan Brestoff

**Affiliations:** Washington University in St. Louis; Washington University School of Medicine; Washington University School of Medicine; Washington University School of Medicine; Washington University School of Medicine; Washington University School of Medicine; Washington University in Saint Louis; University of Texas San Antonio; Washington University in St. Louis; Washington University School of Medicine in St. Louis

## Abstract

B lymphocytes may facilitate chronic inflammation through antibody production or secretion of cytokines, including lymphotoxin (LT)-a_1_b_2_ associated with development of lymphoid tissue. Tertiary lymphoid structures (TLS) characterize human and murine ileitis by suppressing outflow from the ileum. Here, we show that B cell-derived secretory IgA protected against ileal inflammation, whereas B cell-derived LTa guarded against ileitis-associated loss of body mass. We initially hypothesized this protection resulted from formation of TLS that suppressed lymphatic outflow and thereby restrained systemic spread of inflammatory signals, but B cell-selective deletion of LTb did not exacerbate weight loss, despite eliminating TLS. Instead, weight loss driven by the cachectic cytokine TNF was exacerbated when LTa_3_, another ligand for TNF receptors, was selectively neutralized. Thus, B cells’ multi-faceted impact on ileitis includes generating secretory IgA, expressing LTa_1_b_2_ to drive formation of TLS, and producing LTa_3_ for protecting against weight loss in the presence of TNF.

## INTRODUCTION

Local intestinal inflammation is a major feature of Crohn’s disease (CD), one of the most common forms of inflammatory bowel disease (IBD)^1, 2^. While gastrointestinal inflammation is a hallmark, up to 46% of CD patients present with systemic manifestations of disease, such as arthritis and low body mass index^3, 4^. In the gastrointestinal tract, Crohn’s disease involves all layers of the intestine, from the mucosal surface to the underlying mesentery, and most frequently affects the ileum^2^. Beginning with the work of B. B. Crohn, who referred to the disease as regional ileitis before it was named after him, striking alterations in the lymphatic vasculature in CD patients were implicated in disease pathology^5, 6^. However, advances in the field of lymphatic biology werefineeded before in-depth studies on the role of lymphatics were feasible. In the last several years, we and others have identified that B cell-rich tertiary lymphoid structures (TLS) arise adjacent to or within dramatically remodeled mesenteric lymphatic collecting vessels draining the ileum in humans with ileal CD and in the TNF^ΔARE^ mouse model of ileitis^7, 8, 9, 10^. In TNF^ΔARE^ mice, deletion of the AU-rich response element (ARE) prolongs the half-life of tumor necrosis factor-a (TNF) mRNA^11^. The increased TNF, whose expression is initiated by signals from the microbiome, promotes ileitis.^11, 12, 13^ The TLS that form during disease progression obstruct the trafficking of immune cells and molecular cargo to draining lymph nodes and promote both leakage of lymph as well as backflow of lymphatic cargo towards adjacent regions of the intestine.^9^ We are left to wonder whether preventing TLS formation would reduce ileitis. Here, we set out to investigate the mechanisms of lymphatic-associated TLS formation in ileitis and to test their impact on disease progression.

B cells are especially enriched in TLS of humans and mice,^7, 9^ leading us to hypothesize that B cells may play a pivotal role in TLS development in ileitis. B cell production of lymphotoxin (LT) is vital for appropriate secondary lymphoid organ organization,^14, 15^ remodeling of the inflamed lymph node^16, 17, 18^ and TLS organization in cancer,^19^ prompting our curiosity of whether B cells might critically promote formation of TLS that arise along the mesenteric lymphatic vessels in the ileitis of TNF^ΔARE/+^ mice. Furthermore, recent literature that calls for re-examining the role of B cells in IBD^20^ provides a broader context for considering the role of B cells in ileitis. Secretory immunoglobulin [sIg], synthesized by gut-resident plasma cells mainly as dimeric IgA, shifts towards IgG in IBD patients, who often have an aberrant anti-microbial antibody responses.^21, 22, 23, 24, 25, 26, 27^ Whether these alterations in antibody actively contribute to disease pathogenesis or are simply downstream of other inflammatory processes that truly drive disease is somewhat unclear. Additionally, B cells can act as antigen-presenting cells to T cells and produce pro- or anti-inflammatory cytokines to modulate disease severity.^28, 29^

In previous studies, total B cell deficiency did not prevent inflammation in the ileum of TNF^ΔARE/+^ mice^30^ but other features of disease, like TLS formation or systemic manifestations like weight loss were not reported. Thus, we revisited the role of B cells in ileitis, beginning with studies involving B cell deficiency and proceeding to inclusion of additional experimental strategies to refine mechanistic insight. TNF^ΔARE/+^ mice without B cells developed ileitis, as previously reported, but they failed to develop mesenteric TLS and presented with greater loss of body weight and muscle mass compared to TNF^ΔARE/+^ mice with B cells. Deficiency of polymeric Ig receptor (pIgR) in intestinal epithelial cells to reduce IgA in the intestinal lumen modestly exacerbated ileitis but did not influence TLS formation or body weight. On the other hand, B cell-selective deficiency in LTβ or treatment of TNF^ΔARE/+^ mice with LTβR-fc to disrupt B cell-produced LTα_1_β_2_ interfered with TLS development, but did not lead to the additional weight loss observed in the absence of B cells. However, TNF^ΔARE/+^ mice with a B cell-selective loss of LTα, which eliminates their expression of both membrane bound LTα_1_β_2_ and secreted LTα_3_^31, 32^, not only lacked mesenteric TLS but also presented with enhanced loss of lean mass. We go on to reveal that selective blockade of LTα_3_^33^, a ligand that does not bind LTbR but instead binds to TNFRs I and II with high affinity and HVEM with lower affinity^32^, has an independent role in controlling body weight of mice with ileitis in a manner that suggests that it may compete with TNFa to attenuate some of the disease-promoting pathology that result from chronic overproduction of TNFa. Altogether, we identify multiple roles for B cells in ileitis that include a key role in production of locally protective sIg, a role in TLS formation that relies on B cell-derived LTα_1_β_2_, and a highly unanticipated role for B cell-derived LTα_3_ in defending body mass.

## RESULTS

### Characterization of B cells and other immune cells in in the mesentery of Crohn’s patients

**Figure 1:**
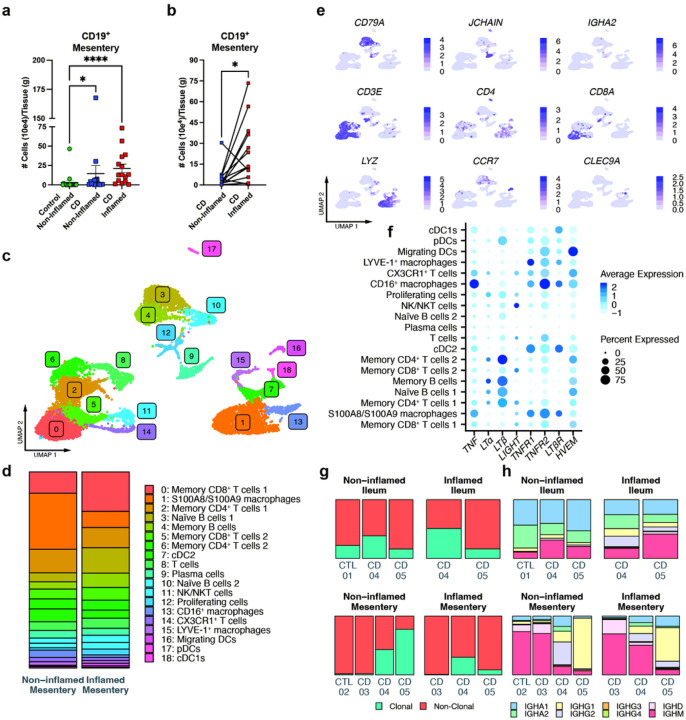
B cells are enriched in the inflamed mesentery of Crohn’s disease patients. **a:** Quantification of the number of CD19^+^ B cells in surgically resected sections of ileal-draining mesentery normalized to the weight of tissue (Control (n = 20), CD non-inflamed (n = 16), CD inflamed (n = 15)). **b:** A subset of the Fig. 1a data to show only CD patients that had matched mesentery draining from non-inflamed ileum and from inflamed ileum, lines connect paired samples from the same patient (n = 13). **c–f:** Single cell RNA sequencing (scRNA-seq) of sorted live, CD45^+^ mesenteric cells from 3 CD patients with matched non-inflamed and inflamed draining mesenteric samples. Uniform Manifold Approximation and Projection [UMAP] plot of mesenteric immune cells **(c)**, the shift in proportion of cells in each cluster comparing non-inflamed and inflamed ileal draining mesentery **(d)**, UMAP plots of a subset of genes used to identify clusters **(e)**, and a dot plot showing average expression of RNA of genes in the TNF/LT family along with their receptors. **g–h:** Single cell BCR sequencing of 3 CD patients and 2 surgical controls of cells from the ileum and mesentery. The proportion of sequenced BCRs in the noninflamed ileum, inflamed ileum, non-inflamed draining mesentery, and inflamed mesentery that are clonal vs. non-clonal **(g)** and what isotype they are **(h)**. Data in a and b represent mean ± SEM. * p < 0.05, **** p < 0.0001. Kruskal-Wallis test **(a)**, Paired t test **(b)**.

Since B cells in the inflamed ileal-draining mesentery of CD patients always localized to TLS^7^, profiling B cells from single cell suspensions of the mesentery would allow us to characterize B cell phenotypes within TLS. Using flow cytometry on such cell suspensions, we confirmed that B cells were significantly enriched in the ileal-draining mesentery of CD patients compared to nearby noninflamed mesentery from the same patients or control ileal-draining mesenteric tissue derived from organ donors or patients with gastrointestinal malignancy (Fig. 1a; gating strategy, Extended Data Fig. 1). Inflamed versus noninflamed regions of the small bowel from Crohn’s patients was determined by a pathologist. Consistent with our past fifindings,^7^ Crohn’s disease-affected mesentery yielded significantly more B cells than nondiseased controls, with the greatest numbers observed in the mesentery associated with inflamed Crohn’s small bowel (Fig. 1a). Focusing on paired samples of inflamed and noninflamed regions, we confirmed that, within the same patient, CD19^+^ B cells in the inflamed regions were elevated in 11 out of 13 CD patients compared to the non-inflamed draining regions (Fig. 1b).

We employed single-cell RNA sequencing [scRNAseq] to characterize B cells and all CD45^+^ cells in the mesentery in 3 CD patients wherein paired noninflamed and inflamed ileal draining mesentery was obtained. A uniform manifold approximation and projection [UMAP] plot of live, CD45^+^ cells recovered revealed 19 clusters corresponding to a range of different immune cells (Fig. 1c). Inflamed mesenteric samples included increased proportions of naïve B cells, in particular, as well as memory B cells (Fig. 1d, e; Extended Data Fig. 2a,b). The top 5 cluster-defining genes are shown in Extended Data Fig. 2c. In addition to the proportional expansion of B cell subpopulations, mesentery from the inflamed ileum of CD patients had an increase in the proportion of proliferating immune cells, compared to non-inflamed ileal-draining mesentery from the same patients (Fig. 1d). While this cluster was not exclusively B cells, a meaningful proportion matched the signature of activation-induced proliferating B cells in TLS. Plasma cells were also detected in small numbers (Fig. 1d). Macrophages were the major producers of TNF among the hematopoietic cells in the mesentery, and they also expressed TNFR1 and TNFR2 most robustly. Memory CD4^+^ T cells and naïve and memory B cells had the highest expression of both LTα and LTβ transcripts (Fig. 1e). Numerically, within the inflamed mesentery, naïve and memory B cells outnumbered memory T cells, underscoring B cells as an important source of LTa and LTb in the region of the mesentery where TLS form. These fifindings reinforced evidence from the literature that B cells may orchestrate TLS generation or maturation via expression of LT proteins. Examination of BCR sequences and Ig isotype expression patterns revealed evidence of oligoclonal expansion of B cell clones in the mesentery in 2 of 3 of the CD patients (Fig. 1g) and these same patients also had an increase in the number of clonal TCRs in their mesentery and ileum (Extended Data Fig. 2d). In those CD patients with an expansion of clonal B cells, the B cells in their mesentery were dominated by production of IgG, not only compared to a control patient, but also to the upstream ileum of those same patients, which were predominately IgA^+^ (Fig. 1g). The one CD patient without B or T cell clonal expansion instead had a large number of naïve B cells in the mesentery suggesting there is heterogeneity in clonal selection in B cell-rich TLSs of CD patients (Fig. 1g,h).

### Absence of B cells in murine ileitis leads to loss of mesenteric TLS but minimal impact on ileal disease

**Figure 2:**
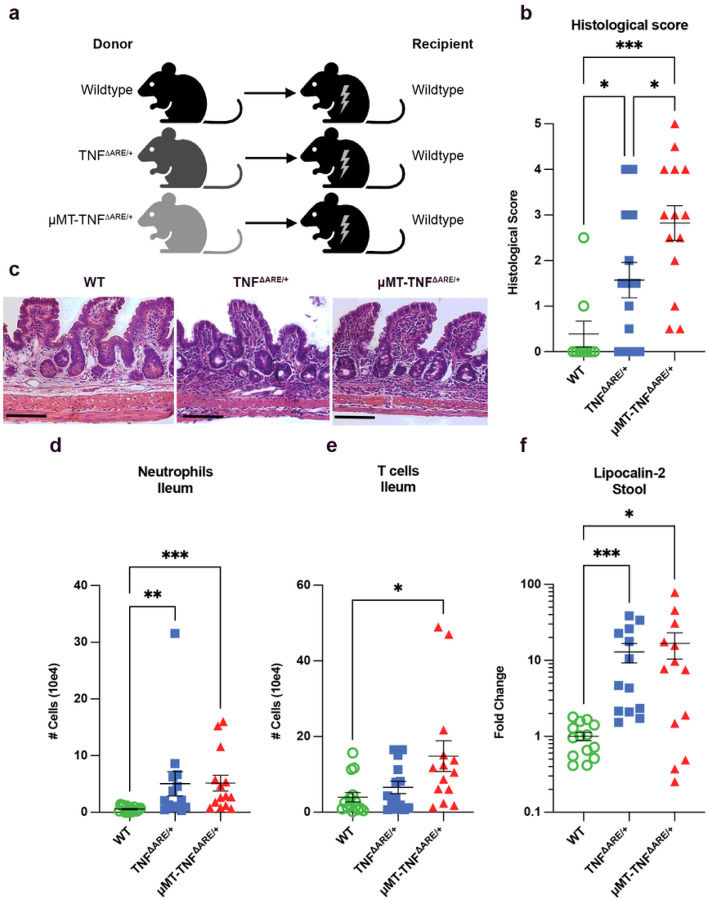
TNF^ΔARE/+^ mice without B cells develop ileitis. WT, TNF^ΔARE/+^, and μMT-TNF^ΔARE/+^ BM recipients 16–17 weeks post-transplant **a:** Schema to describe the bone marrow chimera groups. **b:** Semi-quantitative histological scoring on the distal ileum of BM chimera recipients (Three independent experiments (WT (n=9), TNF^ΔARE/+^ and μMT-TNF^ΔARE/+^ (n = 14)). **c:** Representative H&E pictures of the distal ileum of mice given WT, TNF^ΔARE/+^, and B cell deficient μMTTNF^ΔARE/+^ BM. Scale bars = 150 μm. **d–e:** Flow cytometry on the ileum for neutrophil **(d)** and T cells **(e)** numbers (Three independent experiments, WT (n = 15), TNF^ΔARE/+^ and μMT-TNF^ΔARE/+^ (n = 14)). **f:** Fold change in the amount of lipocalin-2 in the stool of mice normalized to the average amount per WT group per experiment (Three independent experiments, WT (n = 14), TNF^ΔARE/+^ and μMT-TNF^ΔARE/+^ (n = 13)). All data represent mean ± SEM. * p < 0.05, ** p < 0.01, *** p < 0.001. One-way ANOVA with post-hoc Tukey test (b), Kruskal-Wallis test (d–f).

Wild type (WT) recipients given TNF^ΔARE/+^ bone marrow (BM) develop ileitis^30^. To investigate whether and how B cells affect ileitis, we crossed TNF^ΔARE/+^ mice first to CD45.1 mice, so that both CD45.1 and CD45.2 mice in these congenic strains could be used to trace BM cells after transplant. We subsequently crossed the TNF^ΔARE/+^ mice to B cell-deficient μMT mice. We then used WT, TNF^ΔARE/+^, and μMTTNF^ΔARE/+^ mice as bone marrow (BM) donors for lethally irradiated, CD45.2^+^ congenic WT recipients (Fig. 2a). Four months after BM transplant, we quantified ileal inflammation in the recipients using three separate methods. First, we examined the distal 2 cm of the ileum by histology and calculated the extent of inflammation using a semi-quantitative scoring approach (Fig. 2b–c).^34^ Second, we quantified the numbers of neutrophils (Fig. 2d) and T cells (Fig. 2e) in the ileum using the flow cytometry gating strategy in Extended Data Fig. 2a. Finally, fecal lipocalin-2, a sensitive biomarker for murine intestinal inflammation,^35^ was measured by ELISA (Fig. 2f). Ileal inflammation developed to a quantitatively similar extent, with a modest increase in disease when assessed blindly by histological scoring (Fig. 2b), in recipients of B cell-deficient μMT-TNF^ΔARE/+^ BM compared to recipients of TNF^ΔARE/+^ BM, consistent with previous findings.^30^ Infiltration of neutrophils was mostly restricted to the ileum, as gating of neutrophils in the blood (Extended Data Fig. 3b) revealed minimal to no neutrophilia, such that few TNF^ΔARE/+^ and μMT-TNF^ΔARE/+^ mice had increased neutrophils in the proximal portion of the small intestine or in the blood compared to WT mice (Extended Data Fig. 4a,b). Monocytes were increased in the blood of recipients that received TNF^ΔARE/+^ or μMT-TNF^ΔARE/+^ BM compared to those receiving WT BM (Extended Data Fig. 4c), with a shift in the proportion of monocyte subsets towards more Ly6C^lo^ non-classical monocytes (Extended Data Fig. 4d).

Flow cytometry in the blood and ileum confirmed that B cells were missing in mice receiving μMT-TNF^ΔARE/+^ BM (Extended Data Fig. 4e,f). Tissue-resident plasma cells can be radioresistant.^36, 37^ Accordingly, although IgA + plasma cells were reduced in μMT-TNF^ΔARE/+^ BM recipients (Extended Data Fig. 4g), they were not entirely absent, and those remaining were of recipient rather than donor BM origin (Extended Data Fig. 4h)

**Figure 3:**
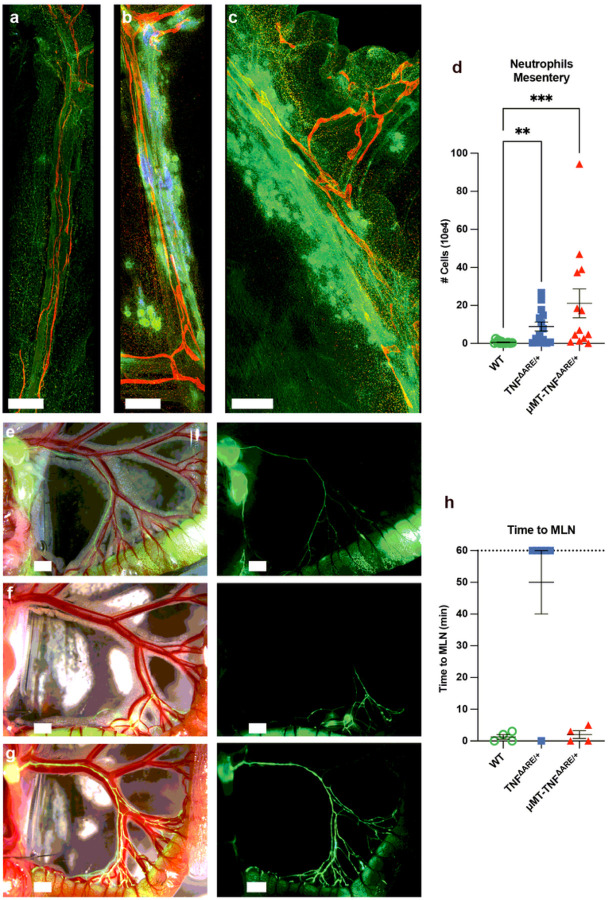
B cells are required for lymphatic remodeling in mice receiving TNF^ΔARE/+^ BM. WT, TNF^ΔARE/+^, and μMT-TNF^ΔARE/+^ BM recipients 16–17 weeks post-transplant. **a-c:** Representative whole mount images (LYVE-1 red, CSF1R green, B220 blue) staining mice that received WT **(a)**, TNF^ΔARE/+^ **(b)** and μMT-TNF^ΔARE/+^ **(c)** BM. **a–c**, scale bars = 500μm. **d:** The number of neutrophils in the mesentery of mice in mice given TNF^ΔARE/+^ and μMT-TNF^ΔARE/+^ BM. (Three independent experiments, WT and TNF^ΔARE/+^ (n = 14) and μMT-TNF^ΔARE/+^ (n = 13)). **e-g:** Representative stereoscope images (left: brightfield and FITC channels, right: FITC channel alone) of the mesentery of anesthetized mice after FITC-dextran injection of 1 to 1.5 μl into the most distal Peyer’s patch, scale bars = 1.5 mm. Mice given WT **(e)**, TNF^ΔARE/+^ **(f)**, and μMT-TNF^ΔARE/+^ **(g)** BM. **h:** Quantification of time to MLN in tracer experiments. (Three independent experiments, WT (n = 4), TNF^ΔARE/+^ (n = 6) and μMT-TNF^ΔARE/+^ (n = 4)). All data represent mean ± SEM. ** p < 0.01, *** p < 0.001. Kruskal-Wallis test (d).

We next examined the mesentery of mice receiving μMT-TNF^ΔARE/+^ BM, because mesenteric inflammation and TLS formation along outflow lymphatic vessels are features of progressive ileal inflammation in this model^9^. In mice receiving TNF^ΔARE/+^ BM, compared to those receiving WT BM (Fig. 3a), many B cell-rich TLS were observed (Fig. 3b). In contrast, although intense CD115^+^ myeloid cell-rich mesenteric inflammation was present in recipients of μMT-TNF^ΔARE/+^ BM, organized TLSs were absent (Fig. 3c). Flow cytometry confirmed marked accumulation of neutrophils in the mesentery of recipients of either TNF^ΔARE/+^ and μMT-TNF^ΔARE/+^ BM compared to those receiving WT BM (Fig. 3d), leading us to conclude that B cells were required for TLS-associated lymphatic remodeling in the mesentery but that the loss of mesenteric TLSs did not prevent accumulation of inflammatory cells in the mesentery.

When we assessed lymphatic outflow through the ileum-draining mesentery after injection of 1–1.5 μl of 2,000 kDa fluorescein isothiocyanate [FITC]-dextran into the most distal Peyer’s patch (Fig. 3e–h), the tracer flowed into mesenteric collecting lymphatic vessels to the mesenteric lymph node [MLN] within Five minutes of injection in recipients bearing WT BM (Fig. 3e, h, Supplementary video 1). However, in 5 out of 6 mice harboring TNF^ΔARE/+^ BM (16–17 weeks after BMT), lymphatic flow to the MLN was not apparent for up to 60 minutes post-injection (Fig. 3f, h, Supplementary video 2), fitting with previous findings that TLSs located along LYVE-1^−^ mesenteric collecting lymphatic vessels obstructed lymph outflow from the ileum^9^. Furthermore, in the one mouse with TNF^ΔARE/+^ BM that had flow of the tracer to the MLN, the dextran tracer still leaked out of the lymphatics, indicating that all mice that received TNF^ΔARE/+^ BM possessed functional lymphatic deficiencies (Supplementary video 3). By comparison, lymphatic flow to the MLN was completely intact in all mice harboring μMT-TNF^ΔARE/+^ BM (Fig. 3g, h), even though the mice receiving μMT-TNF^ΔARE/+^ BM retained ileal inflammation (Fig. 2b–f, Supplementary video 4). These findings confirm that B cells are critical to the development of blocked outflow of ileal lymph in TNF^ΔARE/+^ mice.

### Absence of B cells is associated with more severe systemic weight loss and reduced muscle mass in murine ileitis

**Figure 4:**
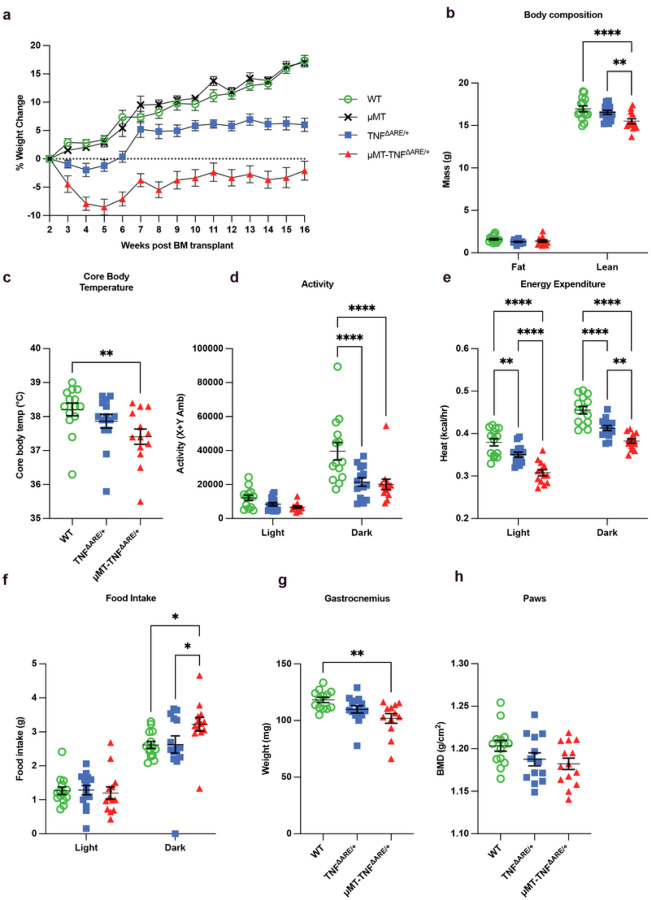
B cells sustain retention of lean mass in mice given TNF^ΔARE/+^ BM. **a:** Weekly percent weight change in mice given WT, μMT, TNF^ΔARE/+^, or μMT-TNF^ΔARE/+^ BM normalized to weight 2 weeks post-transplant (Six independent experiments, WT (n = 24), μMT (n = 9), TNF^ΔARE/+^ (n = 22), μMT-TNF^ΔARE/+^ (n = 23)). **b–f:** Mice given WT, TNF^ΔARE/+^, and μMT-TNF^ΔARE/+^ BM 14 weeks post-transplant. **b:** EchoMRI evaluation of the body composition of mice given WT, TNF^ΔARE/+^, or μMTTNF^ΔARE/+^ BM. **c:** Core body temperature of mice given WT, TNF^ΔARE/+^, or μMT-TNF^ΔARE/+^ BM. **d-f:** Mice were housed in metabolic cages for 24 hours, and activity **(d)**, heat **(e)**, and food intake **(f)** were measured. **g:** Weight of the gastrocnemius muscle in BM recipients. **h:** micro-CT analysis of paws of the bone mineral density. (Three independent experiments WT and TNF^ΔARE/+^ (n = 14), μMT-TNF^ΔARE/+^ (n = 13)). All data represent mean ± SEM. * p < 0.05, ** p < 0.01, *** p < 0.001, **** p < 0.0001. One-way ANOVA with post-hoc Tukey test (c, g, h), Two-way ANOVA (to test if light vs. dark is different in addition to if there are group differences) (d–f).

**Figure 5:**
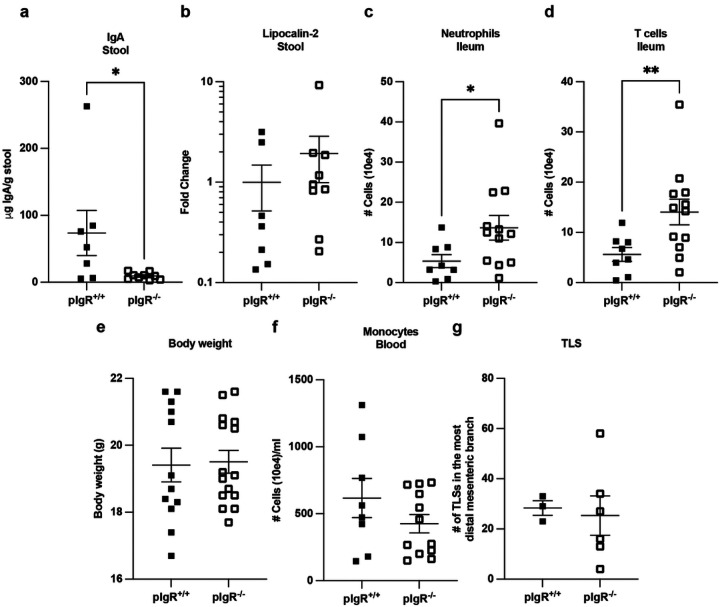
Secretory antibody partially protects against ileal inflammation in TNF^ΔARE/+^ BM recipients, but this protection is limited only to the ileum. Lethally irradiated pIgR^−/−^ mice or pIgR^+/+^ mice given TNF^ΔARE/+^ bone marrow 16 weeks post-transplant. **a**, μg of IgA per gram of stool in pIgR^−/−^ vs. pIgR^+/+^ recipients given TNF^ΔARE/+^ BM. **b**, Fecal lipocalin-2 levels normalized to the average fecal lipocalin-2 amount in the pIgR^+/+^ TNF^ΔARE/+^ BM controls per experiment. (A–B, Two independent experiments, pIgR^+/+^ (n = 7), pIgR^+/+^ (n = 9). **c–d**, Flow cytometry on the ileum shows numbers of neutrophils **(c)** and T cells **(d)**. Three independent experiments, pIgR^+/+^ (n = 8), pIgR^−/−^ (n = 12)). **e**, Total body weight (Four independent experiments, pIgR^+/+^ (n = 12), pIgR^−/−^ (n = 15)). **f**, Number of monocytes in the blood. (Three independent experiments, pIgR^+/+^ (n = 8), pIgR^−/−^ (n = 12)). **g**, Number of TLS along the most distal branch of the mesentery (One experiment, pIgR^+/+^ (n = 3), pIgR^−/−^ (n = 6)). All data represent mean ± SEM. * p < 0.05, ** p < 0.01. Mann-Whitney test for all.

Many CD patients are underweight, and even CD patients with a normal BMI may have loss of muscle mass, a condition called sarcopenia, which seems to arise from a combination of malnutrition, lower activity, and unfavorable protein synthesis or degradation in muscle^38, 39^. TNF^ΔARE/+^ mice were often stunted pre-weaning before the onset of ileal inflammation. Our use of BM chimeras, where all recipients were WT mice that developed normally to adulthood before BM transplant, allowed for a rigorous approach to study the alterations in body composition in response to ileitis disconnected from problems in earlier development. WT mice that received WT BM increased body weight by 17% between 2 and 16 weeks after BM transplant (Fig. 4a) In contrast, mice receiving TNF^ΔARE/+^ BM initially lost weight before experiencing a moderate increase that plateaued 7 weeks later, such that these mice had a mean 6% increase from their initial weight (Fig. 4a). Recipients of μMT-TNF^ΔARE/+^ BM fell behind in weight compared to those getting TNF^ΔARE/+^ BM, exhibited a net loss at 16 weeks post-BM of −2% instead of the gains observed in the other groups (Fig. 4a; Extended Data Fig. 5a). However, B cell deficiency alone without inflammation did not lead to weight loss as μMT BM donors not carrying the TNF^ΔARE/+^ genotype had an increased their weight by 17%, mirroring WT mice (Fig. 5a). We used an MRI-based body composition analyzer to evaluate mice receiving WT, TNF^ΔARE/+^, μMT-TNF^ΔARE/+^ BM at 14 weeks after transplant; fat mass did not significantly change between the groups, but lean mass declined in mice with μMT-TNF^ΔARE/+^ BM compared to both other groups (Fig. 4b).

We then set out to evaluate the whole-body metabolism of these mice 14 weeks after BM transplant by placing them in metabolic cages and observing them over 24 hours. We found that mice receiving μMT-TNF^ΔARE/+^ BM had a decrease in core body temperature compared to those receiving WT BM (Fig. 4c), making it unlikely that the observed weight difference in recipients of μMT-TNF^ΔARE/+^ BM is driven primarily by brown adipose tissue thermogenesis, as the core temperature of mice undergoing thermogenesis should be maintained or increased. Mice are nocturnal and therefore most active during the dark phase; however, mice that received TNF^ΔARE/+^ BM were significantly less active in the dark phase than mice that received WT BM (Fig. 4d). Energy expenditure (heat) was decreased in recipients of either TNF^ΔARE/+^ BM and μMT-TNF^ΔARE/+^ BM during both the light and dark phase compared to those receiving WT BM (Fig. 4e; Extended Data Fig. 5b), although the effect was more pronounced in mice that received μMT-TNF^ΔARE/+^ BM. Contrary to our expectations, food intake in the dark phase was increased in mice bearing B cell-deficient TNF^ΔARE/+^ BM compared to the other groups, indicating that food intake did not explain the increased weight deficit (Fig. 4f; Extended Data Fig. 5c). There was no difference between the groups in water intake (Extended Data Fig. 5d) or in whole-body hydration as assessed by body composition analyses (Extended Data Fig. 5e). The loss of lean mass evident in mice receiving μMT-TNF^ΔARE/+^ BM might reflect a decrease in muscle mass, bone mass, solid organ weight, or a combination of these. Indeed, the weight of the gastrocnemius muscle at 16–17 weeks post-BM transplant was decreased in mice bearing μMT-TNF^ΔARE/+^ BM compared to the other two groups (Fig. 4g). We also measured bone mineral density in the paws of mice 16–17 weeks after BM transplant but found no difference between the groups (Fig. 4h). TNF^ΔARE/+^ mice, in addition to ileitis, also develop rheumatoid arthritis-like disease in their joints^11^, and we observed evidence of arthritis in mice with TNF^ΔARE/+^ BM. When B cells were not present, arthritis worsened; bone porosity was decreased (Extended Data Fig. 5f), while bone surface to bone volume, indicative of bone pitting, was increased (Extended Data Fig. 5g).

These data indicate that systemic features of ileitis, including weight loss and reduced muscle mass, were exacerbated in conditions wherein B cells were lacking. Because these conditions correlated with a loss of TLSs that block lymphatic outflow from the ileum, we began to consider the possibility that the TLS blockade might safeguard against systemic dissemination of inflammatory mediators that originated from the intestine, a concept consistent with recent work that linked valve patency to systemic inflammatory status^40^. However, a rigorous consideration of this concept required that we dissect the role of B cells in ileitis in conditions when B cells were not completely absent.

### Polymeric Ig receptor deficiency in the context of TNF ^ΔARE/+^ ileitis points to a protective role for fecal sIg in local intestinal inflammation but no impact on weight loss

We began our strategy to dissect distinct roles of B cells by first seeking to understand the role of secretory Ig (sIg), which would include IgA transported to the intestinal lumen, in TNF^ΔARE^ driven ileitis. As pIgR is expressed on epithelial cells and controls basolateral translocation of polymeric Ig into the lumen of the intestine, we lethally irradiated pIgR^−/−^ mice or pIgR^+/+^ littermates, reconstituted both genotypes with TNF^ΔARE/+^ bone marrow, and separately housed them after the transplant. The pIgR^−/−^ mice harboring TNF^ΔARE/+^ BM had much less IgA in the stool compared to pIgR^+/+^ TNF^ΔARE/+^ BM recipients (Fig. 5a), although there was still some detectable IgA present, perhaps because, especially in the context of inflammation, immunoglobulin can reach the lumen of the intestine via pIgR independent mechanisms. No difference in fecal lipocalin-2 was detected between the two groups (Fig. 5b). However, in the ileum lamina propria, pIgR^−/−^ TNF^ΔARE/+^ BM recipients compared to pIgR^+/+^ TNF^ΔARE/+^ BM recipients had an increase in inflammatory infiltrating cells with more neutrophils (Fig. 5c) and in T cells (Fig. 5d). These results suggest that sIg is locally protective in the gastrointestinal tract. By comparison, there was no difference in absolute body weight (Fig. 5e) or in the percent weight change at week 16 post-BM. The number of monocytes in the blood, a surrogate for more systemic inflammation, was also similar between the groups (Fig. 5f). The number of TLSs in the most distal mesenteric fat branch—the location where the blood and lymphatic vessels that drain most of the ileum—was also not different between pIgR^+/+^ and pIgR^−/−^ TNF^ΔARE/+^ BM recipients (Fig. 5g). Together, these results indicate that, as anticipated, a protective role of B cells in ileitis is to support plasma cell secretion of sIg. This mechanism is locally protective against inflammation at the intestinal wall, but B cell modulation of body weight, mesenteric TLS organization, and lymphangiogenesis are independent of sIg.

### LTα_1_β_2_ produced by B cells is required for TLS development

As B cell production of LT has been implicated in TLS organization and lymphangiogenesis under inflammatory conditions^16, 17, 18^ and B cells producing LTα and LTβ are present within the inflamed mesentery of CD patients (Fig. 1e) we sought to test if B cell synthesis of lymphotoxins in the TNF^ΔARE/+^ model governed TLS formation. Since the genes for TNFa, LTα, and LTβ are very close to each other within the MHC loci, crossing LTα KO or LTβ KO mice to TNF^ΔARE^ mice was not tractable, as the likelihood of a recombination event would be extremely low^41^. Thus, we instead used mixed BM chimeras where 90% of the donor bone marrow was provided by μMT-TNF^ΔARE/+^ donor marrow and 10% of the bone marrow was supplied by WT, LTα^−/−^, or LTβ^−/−^ donors. Recipients with WT/μMT-TNF^ΔARE/+^ mixed BM should still develop TNF^ΔARE/+^-induced ileitis, but the WT BM supplies B cells that would be exclusively WT. The resulting mice should closely resemble TNF^ΔARE/+^ BM recipients. Recipients of 10%/90% mixtures of LTα^−/−^/μMT-TNF^ΔARE/+^ and LTβ^−/−^/μMT-TNF^ΔARE/+^ BM should also be impacted by TNF^ΔARE/+^ induced ileitis, but for these mixtures, all B cells would lack either LTa or LTb. By contrast, all hematopoietic cells except B cells will primarily (approximately 90%) arise from the μMT-TNF^ΔARE/+^ BM, so the LT deficiency will be B cell specific. LT can signal either as a LTα_3_ homotrimer or a LTα_1_β_2_ heterotrimer; therefore, LTα^−/−^/μMT-TNF^ΔARE/+^ BM recipients will lack both B cell production of LTα_3_ and LTα_1_β_2_, and LTβ^−/−^/μMT-TNF^ΔARE/+^ BM recipients will lose B cell production of LTα_1_β_2_ but will still retain B cell production of LTα_3_. Accordingly, we first examined LTβ^−/−^/μMT-TNF^ΔARE/+^ BM recipients as they lack only one form of LT instead of both.

**Figure 6:**
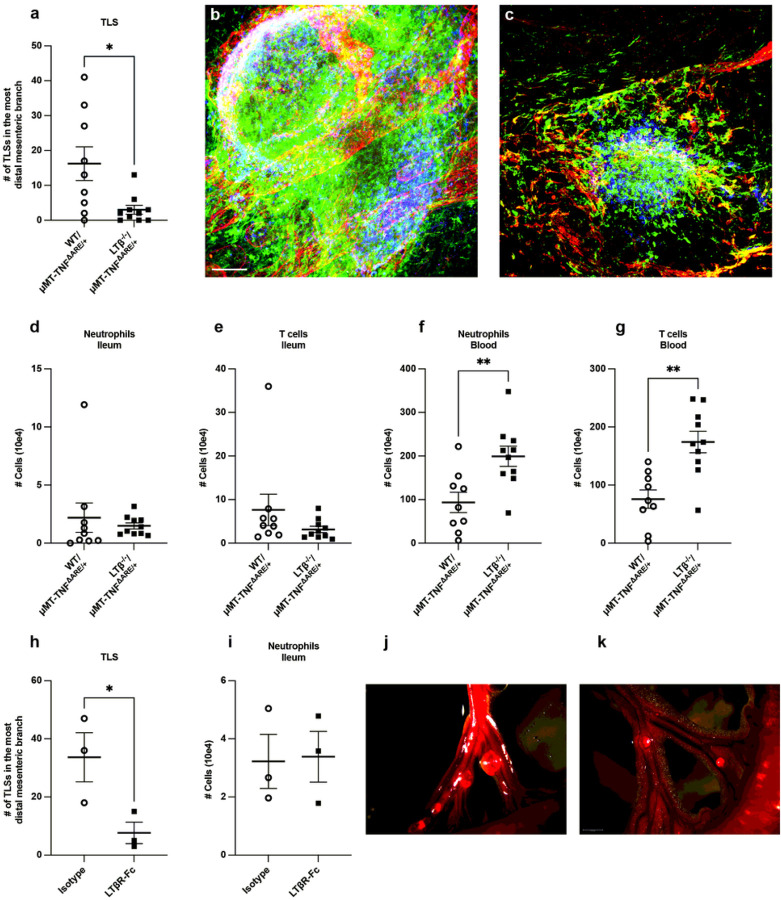
B cell production of LTa_1_b_2_ is required for robust mesenteric TLS in ileitis. **a:** Quantification of the number of TLS in the branch of the mesentery that drains the distal ileum. **b,c:** Representative whole mount images (LYVE-1 red, CSFR1 green, B220 blue) of lymphoid aggregates in the ileal associated mesentery of mice given WT/ μMT-TNF^ΔARE/+^ (b) or LTb^−/−^/μMT-TNF^ΔARE/+^ **(c)** BM. Scale bars, 500 μm. **d,e:** Flow cytometry on the distal ileum of mice that received WT/μMT-TNF^ΔARE/+^ or LTb^−/−^/ μMT-TNF^ΔARE/+^ BM to quantify neutrophils **(d)** and T cells **(e)**. **f,g:** Flow cytometry on the blood of mice given WT/μMT-TNF^ΔARE/+^ and LTb^−/−^/μMT-TNF^ΔARE/+^ BM to quantify neutrophils **(f)** and T cells **(g)**. Two independent experiments were done, comparing WT/μMT-TNF^ΔARE/+^ to LTb^−/−^/μMT-TNF^ΔARE/+^ BM recipients, WT/μMT-TNF^ΔARE/+^ (n = 9), LTb^−/−^/μMT-TNF^ΔARE/+^ (n = 10). h,i: 8 weeks after TNF^ΔARE/+^ BM transplant, mice were dosed weekly with isotype control or LTbR-Fc for an additional eight weeks in one experiment, isotype and LTbR-fc (n = 3 mice in each cohort). **h:** TLS were quantified in TNF^ΔARE/+^ BM recipients given LTbR-Fc in the distal mesentery or isotype control. **i:** Flow cytometry on the ileum of mice given TNF^ΔARE/+^ BM and treated with LTbR-fc or isotype control to quantify neutrophils. **j,k:** Representative stereoscope images of a CD19^cre/+^Td^tomato/+^/μMT-TNF^ΔARE/+^ **(j)** and CD19^cre/+^Td^tomato/+^TNF^fi/fi^/μMT-TNF^ΔARE/+^ **(k)** BM recipient with Td^tomato^ signal overlaid onto a brightfield image. The length of the scale bars represents 1.5 mm. Graphic plots depict mean ± SEM. * p < 0.05, ** p < 0.01. Mann-Whitney tests were used in all graphs of this figure.

At 30 to 32 weeks post-BM transplant, more than a dozen TLS arose in the most distal ileal draining branch of the mesentery of mice that received WT/μMT-TNF^ΔARE/+^ BM (Fig. 6a). Consistent with the morphology of ileitis-associated TLS^9^, these TLS had with lymphatic capillaries that had expanded to wrap around them (Fig. 6b). However, mice that received LTβ^−/−^/μMT-TNF^ΔARE/+^ BM transplants, so that B cells selectively lacked LTb expression, had few TLS present in the distal ileal draining mesentery (Fig. 6a), although some mice did develop occasional undeveloped lymphoid clusters with no evidence of associated lymphomagenesis (Fig. 6c). Both groups of mice had similar neutrophil (Fig. 6d) and T cell (Fig. 6e) Infiltration into the ileum, suggesting that loss of LTb in B cells or consequent loss of TLS did not substantially impact the inflammatory component of ileitis. In the blood, the number of neutrophils (Fig. 6f) and T cells (Fig. 6g) circulating in mice that received LTβ^−/−^/μMT-TNF^ΔARE/+^ BM significantly increased compared to mice given WT/μMT-TNF^ΔARE/+^ BM. Since LTα_1_β_2_ exclusively signals through LTβR, we further tested these finding in the mixed chimera BM transplant setting by dosing with soluble LTβR fused to an Fc domain (LTβR-Fc) to specifically target LTα_1_β_2_. As observed in mice where B cells were engineered to genetically lack LTβ, there was a reduction in TLS number in the ileal draining mesentery in mice given LTβR-fc compared to isotype (Fig. 6h), but no difference in neutrophilic Infiltration into the ileum(Fig. 6i). These data fail to support the hypothesis that mesenteric TLS robustly exacerbate local ileal inflammation, though the loss of TLS may be countered by other ways in which LTb deficiency might impact outcomes.

Because TNF and LT are closely related cytokines, we tested whether B cell production of TNF was important for TLS organization in TNF^ΔARE^ ileitis. We generated CD19^cre/+^Td^tomato/+^ TNF^+/+^and CD19^cre/+^Td^tomato/+^TNF^fi/fi^ mice to generate mixed bone marrow chimeras with μMT-TNF^ΔARE/+^ BM wherein B cells possessed the gene to express TNF or not. We checked the percentage of B cells in the blood of these BM recipients 10 weeks post-BM chimera and found essentially all of the CD19^+^ antibody-stained cells in the blood were CD45.2^+^ and therefore came from the CD19^cre/+^Td^tomato/+^ or CD19^cre/+^Td^tomato/+^TNF^fi/fi^ donor (Extended Data Fig. 5a, b). Furthermore, we observed that an average of 83.7% of the cells stained by an anti-CD19 antibody expressed Td^tomato^ in the CD19^cre/+^Td^tomato/+^/μMT-TNF^ΔARE/+^ mice, but that there was a significant increase in the number of Td^tomato^+ cells in the CD19^cre/+^Td^tomato/+^TNF^fi/fi^/μMT-TNF^ΔARE/+^ BM recipients to 96.7% (Extended Data Fig. 6c). This persisted through the end of the experiment where there was a significant increase in the Td^tomato^+ CD19^+^ B cells (Extended Data Fig. 6d) and IgA^+^ plasma cells (Extended Data Fig. 6e) in mice receiving CD19^cre/+^Td^tomato/+^TNF^fi/fi^/μMT-TNF^ΔARE/+^ BM compared to those receiving CD19^cre/+^Td^tomato/+^/μMTTNF^ΔARE/+^ BM. It seems that, even though the B cells in these mice do not carry a deletion in the AU-rich regulatory element of TNF mRNA and, therefore, are not producing more TNF protein themselves, that there is a cell survival benefit for B cells that cannot make TNF in this context. Additionally, both mice where the B cells could (Fig. 6j) or could not produce TNF (Fig. 6k) still developed well-organized TLS with distinct B cell follicles along the mesenteric lymphatics. Therefore, B cell production of TNF appears dispensable for TLS.

### LTα_3_ produced by B cells protects against excessive weight loss in the context of TNF^ΔARE/+^ ileitis

**Figure 7:**
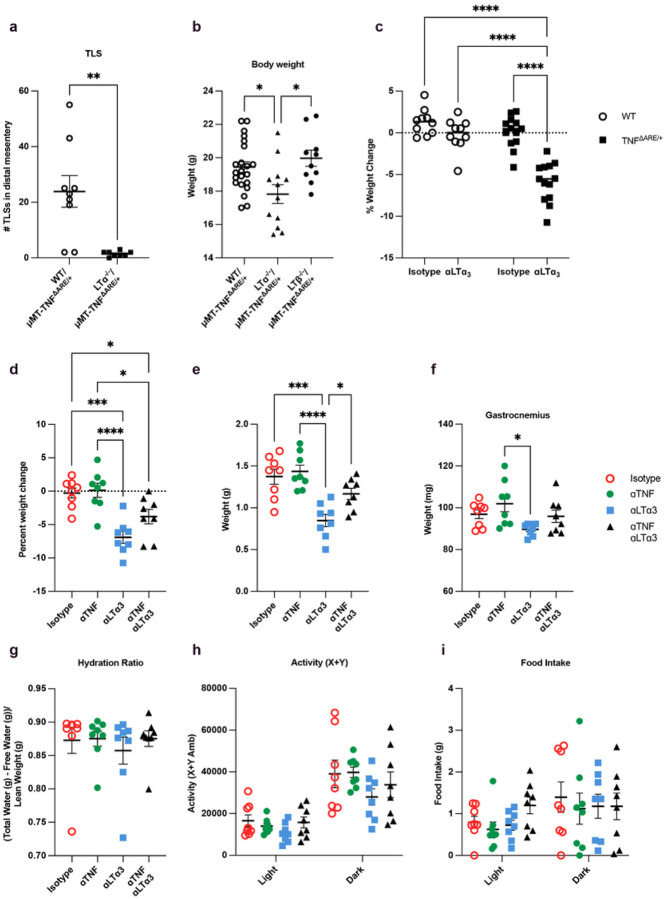
B cell production of LTa_3_ protects against weight loss in ileal inflammation. **A:** Quantification of the number of TLS in mice given LTa^−/−^/μMT-TNF^ΔARE/+^ BM compared to mice that received WT/ μMT-TNF^ΔARE/+^ BM. **b:** Body weight of WT/ μMT-TNF^ΔARE/+^, LTa^−/−^/μMT-TNF^ΔARE/+^ BM recipient mice and LTb^−/−^/μMT-TNF^ΔARE/+^ BM recipient mice at 30 weeks post-BM transplant. **c:** Percent weight change of mice comparing day 0 to day 2 in mice transplanted with WT or TNF^ΔARE/+^ bone marrow 9 weeks post-transplant, then dosed with anti-LTa3 antibody or only isotype. **d:** Percent weight change of mice comparing day 0 to day 2 in mice transplanted with WT or TNF^ΔARE/+^ bone marrow 9 weeks post-transplant, then dosed with anti-LTa3 antibody, anti-TNF antibody, both, or only isotype. **e:** MRI measurements of the body fat composition on day 3. **f:** Weights of the gastrocnemius muscle on day 5. **g–i**, Hydration ratio, activity, and food intake at day 3. All plots mean ± SEM. * p < 0.05, ** p < 0.01, *** p < 0.001, **** p < 0.0001. One symbol represents data from one individual mouse.

We hypothesized that B cell-specific LTα deficiency would phenocopy B cell-specific LTβ deficiency. Like mice given mixed LTβ^−/−^/μMT-TNF^ΔARE/+^ BM, mice that received mixed LTα^−/−^/μMT-TNF^ΔARE/+^ BM did not generate robust TLS in the ileal-draining mesentery when compared to mice given mixed WT/μMT-TNF^ΔARE/+^ BM (Fig. 7a). However, we observed more weight loss in mice given mixed LTα^−/−^/μMT-TNF^ΔARE/+^ BM, where there is a B cell-specific LTα deficiency, compared to mixed bone marrow chimeras where B cells were WT or LTb-deficient (Fig. 7b; Extended Data Fig. 7). This finding was reminiscent of the more severe reduction in body weight we earlier observed in B cell-deficient TNF^ΔARE/+^ BM compared to TNF^ΔARE/+^ BM (Fig. 4a). To further evaluate whether the differences in weight loss between B cells deficient in LTa versus LTb could be attributed to a distinct role for LTa_3_ and LTα_1_β_2_, we turned to a Fc-mutant anti-LTα_3_ antibody that binds and neutralizes LTα_3_ but does not deplete cells producing LTα_3_^33^ While this antibody neutralizes LTα_3_, it has limited ability to bind to the LTα_1_β_2_ heterotrimer *in vitro*, and does not induce splenic architecture abnormalities *in vivo*, unlike dosing with LTβR-Fc^33^. The impact of the anti-LTa3 mAb on body weight was not observed in WT mice. While TNF^ΔARE/+^ BM recipients given anti-LTα_3_ mAb rapidly lost over 5% of their initial body weight after only 2 days, the weight of WT mice receiving the reagent remained steady (Fig. 7c). This result suggested that neutralizing LTa_3_ impacts weight loss only in some contexts, such as a context with TNF signaling was ongoing. To test this idea further, we studied TNF^ΔARE/+^ BM transplanted mice treated with either (i) isotype mAb only, (ii) anti-TNF mAb, (iii) anti-LTα_3_ mAb, or (iv) combined anti-TNF and anti-LTα_3_ mAbs. TNF^ΔARE/+^ BM recipients given anti-LTα_3_ again rapidly lost over 5% of their initial body weight after only 2 days, while isotype-treated and anti-TNF treated TNF^ΔARE/+^ BM recipients maintained their weight, and there was a partial rescue of the anti-LTα_3_ weight loss in mice given both anti-TNF and anti-LTα_3_ (Fig. 7d), suggesting that the biological impact of anti-LTa3 was directly connected to the biological availability of TNF. When the 4 groups of mice were analyzed by MRI, the anti-LTα_3_ treated mice had a significant reduction in the fat mass (Fig. 7e). Additionally, if dosing was extended to 5 days, there was a trend towards a decrease in gastrocnemius weight in TNF^ΔARE/+^ BM-bearing mice treated with anti-LTα_3_ (Fig. 7f). No difference was observed in hydration ratio (Fig. 7g), indicating that the weight loss was not primarily driven by dehydration. Changes in activity (Fig. 7h) or food intake (Fig. 7i) also did not explain the weight loss. Loss of LTα_3_ in B cells or neutralization with anti- LTα_3_ did not prevent absorption, as bomb calorimetric analysis in these conditions did not yield higher caloric retention in feces (Extended Data Fig. 8a,b) and challenge with an oral bolus of triglyceride uncovered no alterations in absorption of fat under steady state (Extended Data Fig. 8c) or in the context of ileal inflammation (Extended Data Fig. 8,d). These data suggest that LTα_3,_ including LTα_3_ produced by B cells protects against inflammation-mediated weight loss in a TNF-driven model of ileitis.

## DISCUSSION

Here, we set out to examine the role of B cells in ileitis, in part motivated by the hypothesis that B cells would have a central role in driving the formation of TLSs that obstruct lymph outflow from the ileum. The concept that B cells might have a central role in the genesis or maturation of TLS through expression of LTα1β114, 15, 16, 17, 18, 19 led us to develop experimental scenarios wherein B cells development was blocked entirely or wherein LTb or LTa were separately and selectively deleted from B cells in TNF^DARE^ mice^11^ that progress to chronic ileitis due to a mutation in TNF mRNA that increases its stability and thus its capacity to promote inflammatory disease. Our analyses included an evaluation of disease severity based on inflammatory criteria, including extent of infiltrated immune cells like neutrophils or lymphocytes, transmural distribution of the infiltrated cells, shedding of lipocalin-2 into the feces, and overall body weight and lean/fat composition. We did not yet consider other important features of the disease, like Paneth cell phenotype or fibrosis, important additional clinical features of human Crohn’s disease. The most striking outcome was that the loss of B cells was associated with an inability to maintain body weight and lean mass as disease progressed, another feature reflected in many humans with Crohn’s disease^3, 4^. By contrast, while loss of gut luminal IgA through epithelial pIgR deficiency led to heightened immune cell Infiltration into the ileum, weight loss did not ensue. It became clear from our data that weight loss and local inflammation did not always track in parallel, an observation that was previously observed in colitis models^42^.

We identified that B cells, in fact, govern the development of TLS that arise at lymphatic valves and inhibit lymphatic outflow in ileitis as it becomes chronic. Deletion of LT_α_ or LT_β_ in B cells, or lack of B cells altogether, was sufficient to halt development of robust TLS, although some mice still had disorganized small foci in regions near where TLS form. However, future studies will be needed to further address the specific impact of TLS on Crohn’s-like ileitis, as the parameters of pathology that we studied, from the local magnitude of inflammatory Infiltration to systemic changes in mass, were not invariably associated with the presence of TLS. In particular, the impact of B cell deficiency of LTb, which blocked TLS formation, was not associated with changes in local inflammation or body weight, though it was associated with a striking increase in the number of immune cells in the circulation.

Unexpectedly, however, deletion of LT_β_ versus LT_α_ in B cells gave rise to divergent results, and the latter more closely reflected outcomes with total B cells deficiency than any other manipulation we made, including studies in pIgR^−/−^ recipient mice that had diminished gut lumen IgA. A previous study revealed that deficiency in LT_β_ versus LT_α_ in RORgt^+^ cells gave rise to different effects on serum IgA, with LTa but not LT_β_ deficiency in RORgt^+^ cells being required for IgA production^43^. Further investigation, in this case, pointed to a role for LT_α1β1_ in induction of T-independent IgA and LTα3 in T-dependent IgA^43^, an outcome where both products generated from the Lta gene were relevant but wherein the presence of LTa_3_ partially masked the impact of losing LT_α1β1_. Indeed, the ability to separate the biology of LT_α3_ from LT_α1β2_ is challenging. In our case, like that of Kruglov et al.^43^, we were led to consider a possible role for LT_α3_ in maintenance of body weight in the face of inflammation by observing discrepancies between phenotypes arising from the loss of LT_α_ versus LT_β_.

Past studies have linked LTα3 to inflammatory responses through its binding of TNFR1 and TNFR2, positioning it to possibly ac redundantly with TNF signaling. For example, it has been argued that LT_α3_ serves as a critical proinflammatory mediator for intestinal inflammation in TNF-deficient mice when epithelial cells lack expression of IBD-associated genes A20 and Abin-1^44^. To support the idea that LT_α3_ drove TNF-independent epithelial cell death, the authors used a mAb that selectively targets LT_α3_^33^. However, it appears that the version of the mAb they used depletes LTa^+^ cells^33^, so while it also neutralizes LT_α3_, the use of the depleting form of themAb would have a broader effect beyond neutralizing LT_α3_, as it would eliminate activated T cells^33^, for instance. We utilized a version of the same mAb with a mutated Fc domain so that it does not deplete LT_α3_^+^ cells, but simply neutralizes secreted LT_α3_^33^. Our findings were in marked contrast to those of Rusu et al.^44^, since in our studies, blocking ${\text{LT}}_{\alpha_{3}}$ led to rapid weight loss in TNF^DARE^ mice. Besides employing different versions of the same reagent with different potential consequences related to cell depletion, it is also possible that the difference in impact between our study versus that of Rusu et al.^44^ could be related to genetic absence of TNF as a ligand for TNFRs in the former model and the role of TNF in driving TNFR-dependent inflammation in our model. That is, it is possible that ${\text{LT}}_{\alpha_{3}}$ engages TNFRs on target cells to promote a less robust inflammatory response than TNF itself. ${\text{LT}}_{\alpha_{3}}$ was, early on, viewed as a partial agonist of TNFRs^45^ and consistently showed more attenuated inflammatory effects in vitro than TNF on the same target cells^46, 47, 48, 49, 50, 51^. Our *in vivo* data raise the possibility, consistent with this historical work, that ${\text{LT}}_{\alpha_{3}}$ propagates inflammation when TNF is absent but serves as a partial brake that dampens TNF/TNFR signaling when both ligands are present. More work is needed to reveal the mechanistic underpinnings of the possible interaction between TNF and ${\text{LT}}_{\alpha_{3}}$.

These findings could contribute to an understanding of why therapeutics like etanercept that neutralize both LT_α_ and TNF failed in IBD, whereas those selectively targeting TNF alone have shown significant efficacy^52^. One of the avenues of future investigation will be a search to better understand how LTa is linked to body weight. The link we uncover in our studies, which we could not explain at the level of caloric intake or metabolic activity, is consistent with several other studies that have found possible regulation of BMI or fat mass to polymorphisms or expression of LT_α_^53, 54, 55^. Our results put a spotlight on ${\text{LT}}_{\alpha_{3}}$ over LT_α1β1_ in that regard.

Overall, in this study, we took a broad look at the role of B cells in ileitis and uncovered disparate areas where they influence the disease in positive or adverse ways, from the protective role conferred locally by IgA production and secretion into the lumen, to their role in TLS formation, and, finally, in their unexpected role for B cell production of LTα_3_ in protection against a systemic manifestation of disease. These findings reinforce the concept that therapeutics to eliminate all B cells non-selectively in IBD patients may be a double-edged sword, where B cell depletion ameliorates some inflammatory aspects of disease progression but worsens others, such as weight less. Furthermore, our findings suggest that targeting specific functions of B cells may enable the development of improved therapeutics to treat IBD. These findings should also provide a solid basis for continued research to better understand the mechanistic basis of pathogenesis in ileitis, and the biological role of TLS in chronic inflammation through the manipulation of B cells.
